# CYP2D6 polymorphisms and endoxifen concentration in Chinese patients with breast cancer

**DOI:** 10.1186/s12885-025-13791-z

**Published:** 2025-03-06

**Authors:** Cong Xue, Wei Yang, Anqi Hu, Caiyun He, Hai Liao, Meiting Chen, Xin An, Shusen Wang, Zhongyu Yuan, Fei Xu, Jun Tang, Haifeng Li, Su Li, Jianyong Shao, Yanxia Shi

**Affiliations:** 1 Department of Medical Oncology, State Key Laboratory of Oncology in South China, Collaborative Innovation Center for Cancer Medicine, Sun Yat-Sen University Cancer Center, 651# Dongfeng Road East, Guangzhou, 510060 P. R. China; 2 Department of Molecular Diagnosis, State Key Laboratory of Oncology in South China, Collaborative Innovation Center for Cancer Medicine, Sun Yat-Sen University Cancer Center, Guangzhou, 510060 P. R. China; 3 Department of Clinical Research, State Key Laboratory of Oncology in South China, Collaborative Innovation Center for Cancer Medicine, Sun Yat-Sen University Cancer Center, Guangzhou, 510060 P. R. China; 4 Department of Breast Cancer, State Key Laboratory of Oncology in South China, Collaborative Innovation Center for Cancer Medicine, Sun Yat-Sen University Cancer Center, Guangzhou, 510060 P. R. China; 5 Guangzhou Targene Biotech Company Ltd, Room 707, Block A, TengFei Street #1 Guangzhou Knowledge City, Huangpu District, Guangzhou, 600700 P. R. China

**Keywords:** Breast cancer, CYP2D6, Endoxifen, Plasma concentration, Tamoxifen

## Abstract

**Background:**

The plasma concentration of endoxifen, the active metabolite of tamoxifen, might be affected by different CYP2D6 genotypes in patients with breast cancer, but solid evidence is still lacking in Asian patients. This prospective study aimed to investigate the relationship between CYP2D6 genetic polymorphisms and endoxifen plasma concentrations among Chinese patients with breast cancer treated with tamoxifen.

**Methods:**

From August 2015 to June 2018, 110 patients with breast cancer were enrolled. CYP2D6 variant alleles and endoxifen plasma concentration were determined using Sanger sequencing and high-performance liquid chromatography-tandem mass spectrometry, respectively.

**Results:**

The most frequent allele of CYP2D6 was *10 (56.4%). The most frequent genotype of CYP2D6 was *10/*10 (33%), *1/*10(28.2%) and *2/*10(14.5%). Sixty-four patients (58.2%) were Normal Metabolizers (NM), while 46 (41.8%) were Intermediate Metabolizers (IM). All patients except two had endoxifen concentrations above the threshold of 5.9ng/ml. The median endoxifen plasma concentrations for patients with CYP2D6 genotypes *1/*2 and *1/10 were higher compared to other genotypes (*p =* 0.012). The median endoxifen plasma concentration was higher in NM than in IM (18ng/ml vs. 13ng/ml, *p* = 0.0077). Patients with CYP2D6*10(T/T) had lower endoxifen concentrations than those with *10(C/T) and *10(C/C) but the difference was not significant. There were no significant differences in adverse events between patients in the NM and IM groups or between patients with the CYP2D6*10 (T/T) genotype and non-*10 (T/T) genotype.

**Conclusion:**

Only CYP2D6 IMs and NMs were identified in this study. Almost all patients had the endoxifen concentrations above the threshold. The endoxifen plasma concentration was lower in CYP2D6 IMs than in NMs, but these variants did not compromise the adverse effects of tamoxifen in Asian patients with breast cancer.

**Trial registration:**

The study protocol was approved by the institutional review boards of Sun YatSen University Cancer Center (Ethics approval number, B201506501,20160115).

**Supplementary Information:**

The online version contains supplementary material available at 10.1186/s12885-025-13791-z.

## Introduction

Breast cancer has become the most common cancer in the world [1]. Hormone receptor positive (HR+) HER2 receptor negative (HER2-) breast cancer accounts for the major part of breast cancer [2]. Endocrine therapy is the most important part of the regimen of these patients, no matter in the early stage or advanced stage. Tamoxifen for 5 years used to be the cornerstone of endocrine therapy, especially in premenopausal patients [3]. The ATLAS and ATTOM studies show the survival of patients with tamoxifen for 10 years is better than those with tamoxifen for 5 years [4, 5]. Nowadays there are more regimens in endocrine therapy to improve the survival of patients with high recurrence risk, for example, aromatase inhibitor (AI) in postmenopausal patients [6]. SOFT and TEXT studies show the additional survival benefits of ovary function suppression (OFS) in premenopausal patients [7, 8]. And MONARCH E study shows abemacilib improved survivals in patients with high recurrence risk [9]. Thus, tamoxifen is now the standard of care in premenopausal and postmenopausal patients with low-risk HR + HER2- breast cancer, or those postmenopausal patients who cannot tolerate AIs.

Four-hydroxy N-desmethyl tamoxifen (endoxifen) is the effective form of tamoxifen, which is biotransformed by CYP2D6 in the liver [10]. CYP2D6 is a highly polymorphic gene with more than 100 allelic variants [11–13]. Some of the alleles have reduced activities (such as *41, *10) or even are nonfunctional (such as *3, *4, *5), leading to a decreased production of endoxifen [14]. Based on the clinical pharmacogenetics implementation consortium (CPIC) guideline [15], each allele of CYP2D6 has a corresponding activity value ranging from 0 to 1. For example, the value of allele *3 is 0, which means no function; the value of *10 is 0.25, which means reduced function. The sum of all alleles is called CYP2D6 activity score (AS), which divides CYP2D6 into four phenotypes: patients with a score above 2 are ultrarapid metabolizers (UM), patients with a score above 1 to 2 are normal metabolizers (NM), those with a score above 0 to 1 are considered intermediate metabolizers (IM), and those with AS of 0 are poor metabolizers (PM). Some studies show that the survivals of patients with PM or specific alleles treated with tamoxifen are poorer than others [16–22]. In some literature, the explanation comes from the lower endoxifen plasma concentration [22–25]. The threshold of endoxifen was reported 5.9 ng/ml [26] and 5.2 ng/ml [27]. About 30% of Caucasian patients, mostly with CYP2D6 PM, had endoxifen concentrations below the suggested threshold [25, 28]. But the results are conflicting [29–34]. Until now there is no consensus reached about the detection of CYP2D6 genotypes and endoxifen concentrations in patients treated with tamoxifen. According to CPIC, patients with PM should switch to OFS + AI or receive a double dosage of tamoxifen [15].

Nevertheless, the distributions of CYP2D6 are quite different among different populations. For example, The CYP2D6 *4 allele is frequently observed in Caucasians, whereas Chinese patients are more commonly associated with the *10 alleles [35, 36]. Chinese patients with *10 (c.100 C > T) T/T have worse survivals than those with C/T or C/C [37, 38]. However, there are no studies that revealed the relationship between CYP2D6 genotypes and endoxifen concentrations in Chinese patients. What’s more, there is no data referring to the CYP2D6 AS or phenotype metabolizers in Chinese patients.

Therefore, this prospective observational study aimed to investigate the relationships between the CYP2D6 polymorphisms and plasma concentration of tamoxifen/endoxifen in Chinese patients with breast cancer treated with tamoxifen.

## Materials and methods

### Patients

From 2015 Aug to 2018 Jun, there were 2469 patients newly diagnosed with HR + breast cancer treated with tamoxifen at Sun Yat-Sen University Cancer Center (SYSUCC). Among these, patients were excluded based on the following criteria: (1) initial adjuvant endocrine therapy including OFS (*N* = 1200); (2) refusal to be enrolled or unwilling to offer blood sample (*N* = 936); (3) without complete follow-up data or adverse events (AEs) recorded in the patients’ medical record (*N* = 223). Finally, 110 patients were included in this study. Because it was an observational study (no intervention was performed), patients with stage IV disease and/or with HER2-positive status were enrolled.

The study protocol was approved by the institutional review boards of SYSUCC (Ethics approval number, B2015-065-01). Written informed consent was obtained from all patients before their enrollment in this study. Comorbidity and concurrent medication that might influence CYP2D6 activity (venlafaxine, quinidine, diphenhydramine and cimetidine) were retrospectively recorded from the medical record.

### CYP2D6 genotyping assay

Genomic DNA was extracted and purified from 300 µL of peripheral blood using a column-based DNA isolation kit (DP 348, TIANGEN Biotech Beijing Co., Ltd. Beijing, China). DNA was amplificated using Probe qPCR Mix with UNG (RR392A, TAKARA, Biomedical Technology Co., Ltd. Kyoto, Japan). DNA sequencing was performed with Sanger sequencing systems (ABI 3500XL, Thermo Fisher Scientific Inc. Massachusetts, United States) [39]. The CYP2D6 genotyping panel included the no-function variants CYP2D6*4 (rs3892097) and CYP2D6*5 (Exon 9 deletion), the decreased-function variants CYP2D6*10 (rs1065852), CYP2D6*14 (rs5030865) and CYP2D6*41 (rs28371725), and the increased-function variant CYP2D6*2 (rs1135840). Without detection of any of the above variant alleles, the genotype was defined as CYP2D6*1/*1 (Details in Supplementary material).

We sum up the AS of CYP2D6 alleles on each patient based on CPIC. Further CYP2D6 metabolizer status was divided into different phenotypes according to the AS [15]. That were three categories of CYP2D6 in our study: genotypes (for example *1/*2), AS (for example 0, 2) and phenotypes (for example UM/NM).

### Endoxifen plasma concentration

Blood samples were collected from each patient when she had taken tamoxifen for at least 30 days. The blood sample was collected five hours after taking tamoxifen. Plasma concentrations of tamoxifen and endoxifen were measured using a high-performance liquid chromatography-tandem mass spectrometry (HPLC-MS/MS, API 2000) assay method [40]. Ethoxybenzamide was used as the internal standard. Standard curve and quality control were established according to tamoxifen or endoxifen and IS ratio. The concentration range of the standard curve was 10-500ng /ml, and the concentration of quality control was 30ng/ml (low), 250ng/ml (medium) and 400ng/ml (high). The concentration of the drug was calculated and reported according to the standard curve.

### AEs

AEs were primarily collected prospectively during patient enrollment. However, given that some patients had been on tamoxifen for certain duration, certain AEs were documented retrospectively from medical records. Only AEs supported by test results in the medical records were recorded. Therefore, liver dysfunction, dyslipidemia, and gynecological events were collected [41–43]. Liver dysfunction and dyslipidemia events were defined as follows: (1) an elevation in liver function tests or lipid levels when baseline values are within normal ranges; (2) a significant deterioration, characterized by an increase of more than twice the baseline level or more than five times the upper limit of normal, when baseline values are mildly elevated. The indicators collected included alanine transferase (ALT), aspartate aminotransferase (AST), alkaline phosphatase (ALP), glutamyl transpeptidase (GGT), total bilirubin (TBIL), triglyceride (TG), total cholesterol (TC), lysophosphatidic acid [LP(a)], high density lipoprotein (HDL), low density lipoprotein (LDL) and non-high-density lipoprotein (NHDL). Gynecological events were defined as endometrial thickening, uterine fibroids, ovarian cysts, adenomyosis, and cervical canal cysts diagnosed by gynecological ultrasound. All these events should appear at least 1 month after endocrine therapy that we assumed were related to tamoxifen.

### Statistical analysis

Analyses were performed with Prism 9 version 9.3.1. P-values of < 0.05 were considered significant. Categorical variables were expressed as frequencies and compared using Pearson’s χ2 test. Continuous variables were expressed as mean ± SD and analysis of variance (ANOVA), Fisher’s exact test, Chi-square test or t-test was performed for comparison of the data. Post hoc (t-test for two columns or Tukey’s honest significant difference analysis for more than three columns) was performed. Duration of TAM was calculated from the date of beginning tamoxifen to the date of relapse or death, switching to other regimens or completing endocrine regimen, or censoring of follow-up.

Hardy–Weinberg equilibrium (HWE) was evaluated using the χ2 test, with the threshold for HWE defined as *p* > 0.05.

## Results

### Baseline characteristics

There were 110 patients enrolled in our study. Until January 14, 2024, the median follow-up time was 70.5 months (range, 1-157.5 months). The median interval between tamoxifen plasma concentration detection and the first prescription of tamoxifen was 2.32 months (range, 0.93–43.7 months). Median tamoxifen usage was 26.13 months (range, 1-102.1 months). Basic characteristics were listed in Table 1. The median age was 45.5 years (range, 26–64 years). Eighty-four patients were premenopausal (76.4%). Eighty-one patients had phase I -II diseases (73.6%). In 100 patients with adjuvant endocrine therapy, all were treated with tamoxifen upfront. During the follow-up, nineteen of them added OFS with tamoxifen (19%); thirteen patients (13%) transferred to toremifene (TOR) ± OFS; sixteen patients (16%) transferred to AI ± OFS; thirteen patients (13%) firstly transferred to TOR ± OFS then AI ± OFS. The rest forty-nine patients (49%) were treated with tamoxifen monotherapy all the time. No participant was found to be using a strong CYP2D6 inhibitor concurrent with tamoxifen.

**Table 1:** Characteristics of patients

Characteristics	ALL	IM	NM	
	No.(%)	*n* = 46(%)	*n* = 64(%)	*p*
Age, years				
Median(range)	45.5(26–64)	46(29–53)	45(26–64)	0.495
Menopause status				0.653
Premenopausal	84(76.4)	34(73.9)	50(78.1)	
Postmenopausal	26(23.6)	12(26.1)	14(21.9)	
Clinical stage				0.997
I	32(29.1)	13(28.3)	19(29.7)	
II	49(44.5)	21(45.6)	28(43.7)	
III	19(17.3)	8(17.4)	11(17.2)	
IV	10(9.1)	4(8.7)	6(9.4)	
Comorbidities^*^				> 0.999
Yes	12	5	7	
No	98	41	57	
ER status				0.570
Positive	107(97.3)	44(95.7)	63(98.4)	
Negative	3(2.7)	2(4.3)	1(1.6)	
PR status				> 0.999
Positive	101(91.8)	42(91.3)	59(92.2)	
Negative	9(8.2)	4(8.7)	5(7.8)	
HER2 status				0.830
Positive	31(28.2)	14(30.4)	17(26.5)	
Negative	78(70.9)	32(69.6)	46(71.9)	
Unknown	1(0.9)	0(0)	1(1.6)	
Adjuvant TAM endocrine therapy(*N* = 100)				0.601
TAM	49(49)	19(45.2)	30(51.7)	
Switch to TAM + OFS	9(9)	3(7.1)	6(10.4)	
Switch to TOR ± OFS	13(13)	8(19.1)	5(8.6)	
Switch to AI ± OFS	16(16)	6(14.3)	10(17.2)	
Switch to TOR ± OFS then AI ± OFS	13(13)	6(14.3)	7(12.1)	
Duration of TAM, months Median (range)		20.9(1-102.1)	27.0(1.03–97.4)	0.787

Abbreviations ER, estrogen receptor; PR, progesterone receptor; TAM, tamoxifen; OFS, ovary function depression; TOR, toremifene; AI, aromatase inhibitor

^*^ Comorbidities were defined as hypertension, diabetes or other diseases existing during the course of breast cancer

### The genotypes, AS, and phenotypes of CYP2D6 and the plasma concentration of tamoxifen and endoxifen

The population genotype distributions confirmed to HWE (*p* > 0.05).

In our study, CYP2D6 genes were all detected as diploid. CYP2D6*10 was the most frequent allele (56.4%), followed by *1 (21.4%) and *2 (14.1%). The most frequent genotype of CYP2D6 were *10/*10 (*n* = 33, 30%), *1/*10 (*n* = 31, 28.2%) and *2/*10 (*n* = 16, 14.5%).

According to CPIC, we divided them into different groups of total AS. The most frequent AS was 1.25 (*n* = 47,42.7%), including those with *1/10 and *2/*10. Following was 0.5 (*10/*10, *n* = 33, 30%) and 2 (*1/*1, *1/*2 and *2/*2, *n* = 14, 12.7%).

Finally, the patients were divided into different CYP2D6 phenotypes. According to CPIC, we found there were only IM (AS below 1, *n* = 46, 41.8%) and NM (AS above 1 to 2, *n* = 64, 58.2%) in our patients. No UM (AS above 2) or PM (AS = 0) were found in our study.

The median concentration of tamoxifen and endoxifen was separately 225.5 ng/ml (range, 69.4–772 ng/ml) and 16.1 ng/ml (range, 2.83–59.5 ng/ml). The median metabolic ratio of endoxifen from tamoxifen (MR_E/Tam_) was 0.071 (range, 0.026–0.160). All patients had endoxifen concentrations above the threshold (5.9ng/ml) except two patients (2.83ng/ml and 5.69 ng/ml).

Table 2 showed the concentration of tamoxifen and endoxifen (ng/ml) among different CYP2D6 polymorphisms, including the genotypes, AS, and phenotypes. The concentration of tamoxifen was similar in different genotypes, AS, or phenotypes (*p* all > 0.05).

**Table 2:** The median concentrations of Tamoxifen and Endoxifen (ng/ml), and metabolic ratio of Endoxifen from Tamoxifen (MR_e/Tam_) among different CYP2D6 polymorphisms

CYP2D6 Genotype	*n* = 110 (%)	tamoxifen	endoxifen	MR_E/Tam_
		*p* = 0.74	*p* = 0.012	*p* = 0.0007
*1/*1	3 (2.7)	167(-65-537)	18(-1.3-41)	0.076(0.034–0.14)
*1/*2	8 (7.3)	222(159–364)	24(18–27)	0.11(0.071–0.12)
*1/*10	31 (28.2)	289(237–334)	23(21–31)	0.089(0.082–0.11
*1/*14	1 (0.9)	167	13	0.077
*1/*41	1 (0.9)	133	18	0.13
*2/*2	3 (2.7)	174(-58-385)	13(-6.6-28)	0.065(0.018-0.10)
*2/*10	16 (14.5)	195(175–229)	16(13–18)	0.073(0.065–0.088)
*2/*41	1 (0.9)	174	12	0.071
*5/*10	5 (4.6)	205(112–394)	12(7.2–17)	0.053(0.055–0.070)
*10/*10	33 (30)	249(224–332)	13(13–20)	0.063(0.055–0.070)
*10/*41	6 (5.5)	207(46–490)	15(11–23)	0.083(0.044–0.12)
*14/*41	1 (0.9)	224	11	0.048
*41/*41	1 (0.9)	202	12	0.059
*Activity Score (genotype)*	*n* *= 110* (%)	*tamoxifen*	*endoxifen*	*MR*_*E/Tam*_
		*p* = 0.795	*p* = 0.106	*p* = 0.0009
0.25(*5/*10)	5 (4.6)	205(112–394)	12(7.2–17)	0.053 (0.024–0.084)
0.5(*10/*10)	33 (30)	249(224–332)	13(13–20)	0.063 (0.055–0.070)
0.75(*10/*41)	6 (5.5)	207(46–490)	15(11–23)	0.083 (0.044–0.12)
1(*14/*41 and *41/*41)	2 (1.8)	213(73–353)	11(3.8–19)	0.054 (-0.016 -0.12)
1.25(*1/*10 and *2/*10)	47 (42.7)	229(222–291)	22(19–26)	0.082 (0.079–0.097)
1.5(*1/*14, *1/*41 and *2*41)	3 (2.7)	167(104–212)	13(7.2–21)	0.077 (0.0098-0.18)
2(*1/*1, *1/*2 and *2/*2)	14 (12.7)	208(169–301)	19(15–24)	0.078 (0.070–0.10)
*CYP2D6 phenotype (AS)*	*n* * = 110 (%)*	*tamoxifen*	*endoxifen*	*MR*_*E/Tam*_
		*p* = 0.358	*p* = 0.0077	*p* < 0.0001
IM (0.25, 0.5, 0.75 and 1)	46 (41.8)	238 (226–316)	13(13–18)	0.061 (0.057–0.071)
NM (1.25, 1.5 and 2)	64 (58.2)	218 (219–276)	18(18–24)	0.081 (0.081–0.095)

Notes The concentrations of tamoxifen and endoxifen, and MR_e/Tam_ are presented as median (95% CI)

Abbreviations IM, intermediate metabolizer; NM, normal metabolizer

The concentration of endoxifen and MR_E/Tam_ was significantly affected by different CYP2D6 genotypes (*p* = 0.012 and *p* = 0.0007, separately). In the post hoc analysis, we found the concentrations of endoxifen were significantly different in the groups *1/*10 vs. *2/*10 (*p* = 0.033) and *1/*10 vs. *10/*10 (*p* = 0.014). Similarly, in the post hoc analysis of MR_E/Tam_, the difference mainly came from the group *1/*10 vs. *10/*10 (*p* = 0.0005).

**Fig. 1:**
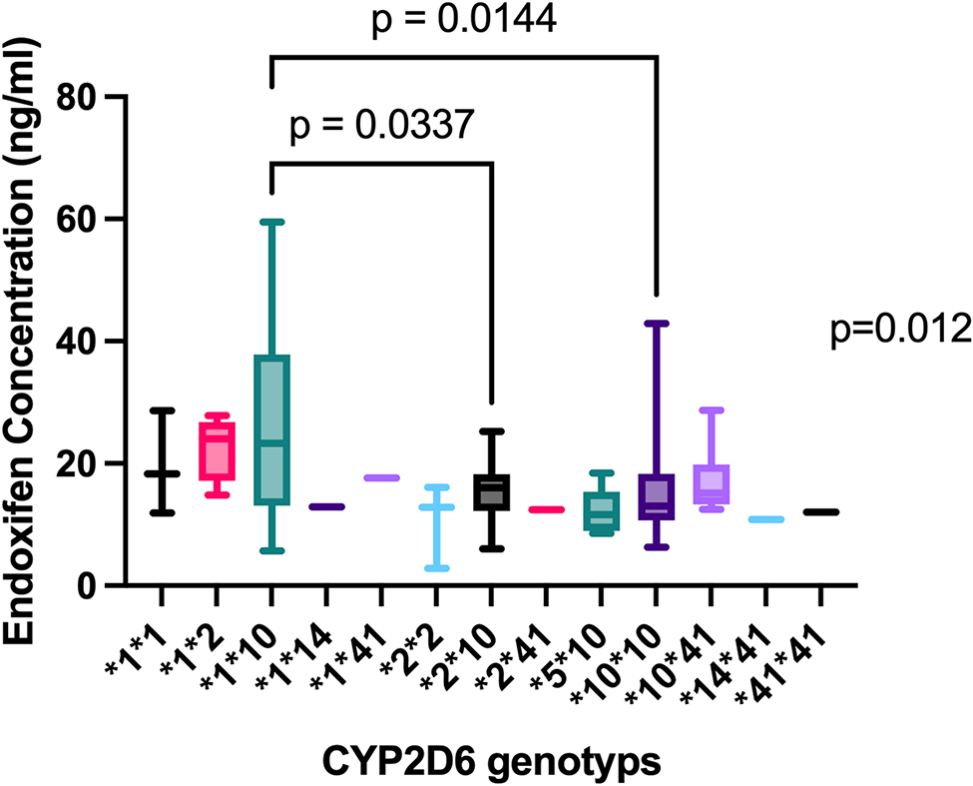
Endoxifen concentration in CYP2D6 genotypes

The median endoxifen concentration was highest in CYP2D6 genotype *1/*2 (24ng/ml, 95%CI 18–27 ng/ml) and *1/*10 (23ng/ml, 95%CI 21–31 ng/ml, Fig. 1). Patients with genotype *1/*10 were with higher median endoxifen concentrations when compared with patients with *10/*10 (*p* = 0.0144) and with *2/*10 (*p* = 0.0337).

Endoxifen concentration was not significantly different among different AS groups (*p* = 0.106). However, MR_E/Tam_ was significantly affected by different CYP2D6 AS (*p* = 0.0009). In the post hoc analysis of MR_E/Tam_, the difference existed between group 0.5 vs. 1.25 (*p* = 0.0015).

**Fig. 2:**
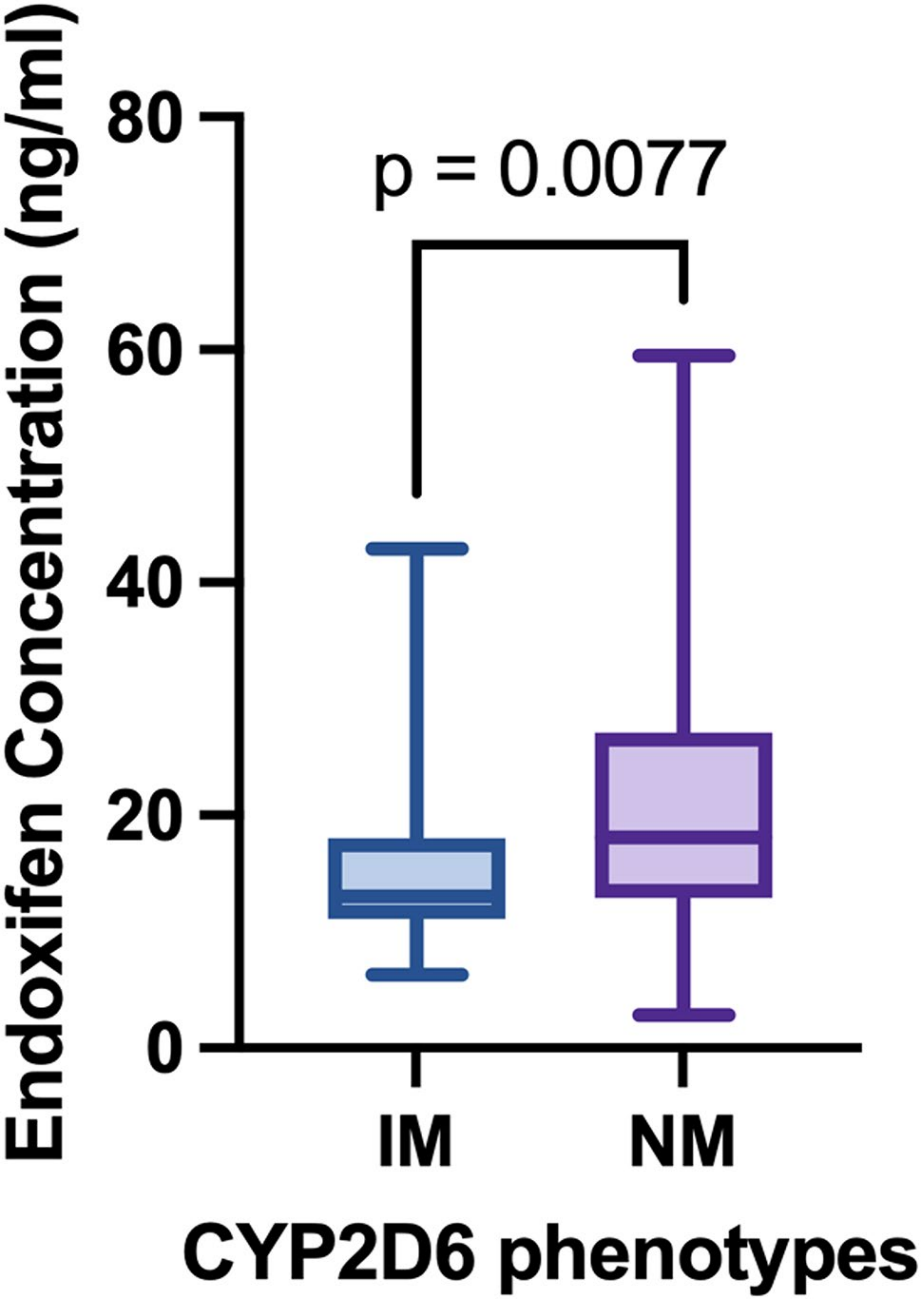
Endoxifen concentration in CYP2D6 phenotypes. Abbreviations IM, intermediate metabolizers; NM, normal metabolizers

And the median endoxifen concentration was higher in CYP2D6 phenotype NM (18ng/ml, 95%CI 18–24 ng/ml) than in IM (13ng/ml, 95%CI 13–18 ng/ml, *p* = 0.0077, Fig. 2). A similar situation was found with MR_E/Tam_ (*p* < 0.0001).

The plasma concentration of endoxifen was separately analyzed between CYP2D6 genotype *10 (c.100 C > T) T/T (*n* = 33) and others (C/C and C/T, *n* = 77). We found the median concentrations of endoxifen were lower in patients with *10 T/T than those with C/T or C/C but the difference was not significant (13 vs. 17 ng/ml, *p* = 0.101). When we divided the patients into three groups: *10 T/T (*n* = 33), C/T (*n* = 58), and C/C (*n* = 19), the median concentrations of endoxifen were still lower in patients with *10 T/T than those with C/T or C/C but the difference was still not significant (13 vs. 17 vs. 16 ng/ml, *p* = 0.146).

The median plasma concentration of endoxifen was similar between premenopausal and postmenopausal patients (16 vs. 15 ng/ml, *p* = 0.709).

### The genotypes and phenotypes of CYP2D6 and the adverse effects

At baseline, 3 (6.5%) patients in the IM group and 7 (10.9%) patients in the NM group exhibited mildly abnormal serum liver function. Fourteen (30.4%) of 46 patients in the IM group and 22 (34.4%) of 64 patients in the NM group had liver dysfunction events. There was no significant difference between IM and NM groups (*p* = 0.686). Endoxifen concentration was not associated with liver dysfunction (*p* = 0.3225). Detailed data were presented in Table S1.

At baseline, 8 (17.4%) patients in the IM group and 15 (23.4%) patients in the NM group exhibited mildly dyslipidemia. Thirty-one (67.4%) of 46 patients in the IM group and 48 (75.0%) of 64 patients in the NM group had dyslipidemia events. There was no significant difference between IM and NM groups (*p* = 0.399). Endoxifen concentration had a trend of association with dyslipidemia (*p* = 0.091). Detailed data were presented in Table S1.

Sixteen (34.8%) of 46 patients in the IM group and 23 (35.9%) of 64 patients in the NM group had gynecological events. There was no significant difference between IM and NM groups (*p* > 0.999). Endoxifen concentration was not associated with gynecological events (*p* = 0.398).

When we divided the patients into CYP2D6 *10 T/T and non-*10 T/T, no significant difference in risks of adverse effects (liver dysfunction events, dyslipidemia events, and gynecological events) was found (*p* = 0.599, > 0.999 and 0.437).

To sum up, the AEs were similar between different CYP2D6 polymorphisms.

### The phenotypes of CYP2D6 and events

At the date of January 14, 2024, 23 patients had disease progression from tamoxifen therapy, 8 of them were IM and the rest were NM. In them, there were eight patients with stage IV disease (all were NM). Five patients with stage III disease (all with distance metastasis), and 4 were NM. Eight patients with stage II disease (one with contralateral breast cancer, one with regional relapse, two with distance metastasis and four with secondary primary tumors including lung cancer, colon cancer, pancreatic cancer and glioblastoma), 6 were NM. Two patients with stage I disease (one with distance metastasis and one with contralateral breast cancer), both were IM. Four patients passed away because of breast cancer. More time was needed for further analysis.

## Discussion

Previous studies on Chinese patients have primarily focused on the CYP2D6 *10 allele and non-*10 allele, with limited investigation of other alleles [38, 44]. In this study, we expanded the detection scope to include several most frequent alleles of CYP2D6 and described the CYP2D6 genotype distribution. To our knowledge, this is the first study to investigate the relationship between CYP2D6 polymorphisms and endoxifen concentration in Chinese patients with breast cancer receiving tamoxifen. When CYP2D6 was divided into different phenotypes in the study, only IM and NM were identified. This is in contrast to the Caucasian population where UM and PM are also present [14, 16]. Finally, this study found that the endoxifen concentration was higher in patients with CYP2D6 NM than those with IM, which was consistent with previous studies [23–25]. This study revealed that most patients had endoxifen concentrations exceeding the threshold value of 5.9ng/ml, which could partly account for the lack of significant differences in survival and adverse reactions between patients with CYP2D6 IM and NM.

Despite numerous studies focusing on the relationship between tamoxifen metabolite concentrations, tamoxifen efficacy, and CYP2D6 polymorphisms, the correlation has yet to be confirmed due to variations in enrollment, detection methods, and other factors [24, 29, 33, 45, 46]. A meta-analysis of 29 studies comprising 13,001 patients showed that mean endoxifen concentrations were significantly lower in PM compared to UM. However, in the majority of these studies, PM status did not have a significant impact on clinical outcomes [47]. These findings suggested that CYP2D6 polymorphisms may partly influence endoxifen concentration, but their impact on tamoxifen efficacy is not significant [47]. Since the current study did not identify any PM cases, and nearly all cases were with enough endoxifen concentrations above a threshold value, tamoxifen should be effective in treating Chinese patients with breast cancer.

CYP2D6 *10 was most prevalent in Asian populations. Previous studies showed that patients with CYP2D6 *10 T/T had lower serum 4OHtam concentrations and worse survival outcomes compared to patients with C/C and C/T [21, 37, 38]. A meta-analysis demonstrated that CYP2D6 *10 polymorphisms have an impact on the pharmacokinetics of tamoxifen in patients with breast cancer of Asian ethnicity [44]. However, in this study, no significant difference in endoxifen plasma concentration was found between CYP2D6 *10 and non-*10, or between *10 T/T, C/T, and C/C. Regarding adverse effects, Zhou and He reported that patients with UM treated with tamoxifen had a higher risk of AEs and were more likely to discontinue tamoxifen [20, 48]. Nevertheless, in this study, there was no significant difference in the incidence of adverse effects between patients with IM and NM, or between patients with CYP2D6 *10/*10 (c.100 > T) and non-*10/*10 (c.100 > T), which is consistent with another study conducted in Asian patients [31].

The inconsistent findings of the aforementioned studies may be attributed to several factors. Firstly, the categorization of patients based on the *10 and non-*10 division, or other similar classification methods, was imprecise and variable, leading to a potential dilution of the modest significant difference. Secondly, even in patients with *5/*10, which had the lowest AS in our study, the IM phenotype exhibited a median endoxifen concentration of 12 ng/ml, surpassing the threshold of 5.9 ng/ml. This suggests that even one functional CYP2D6 allele can produce adequate endoxifen. Lastly, patients with CYP2D6 UM displayed a higher frequency of adverse effects, causing them to postpone or suspend tamoxifen therapy, or switch to alternative endocrine therapies. This highlights the crucial role of adverse effects in compromising the detection of CYP2D6 genotype and endoxifen concentration.

We assumed the threshold value of endoxifen (5.9ng/ml) was important in Caucasians because 30% of patients were with CYP2D6 PM. Recently Africans (Zimbabwean and South African) also reported that half of the patient population were with PM. Endoxifen concentrations of patients with PM were significantly lower than those with UM/NM/IM [49, 50]. As approximately 50% of the Asian population was with CYP2D6 IM, endoxifen concentrations were rarely reported below the threshold. Zembutsu et al. reported the mean plasma endoxifen concentrations were 9.3ng/ml in Japanese patients with CYP2D6 IM taking TAM 20 mg/d [51], similar to our findings.

Currently, there are several confirmed regimens for improving DFS in patients with breast cancer, including prolonged tamoxifen duration [4], switching to AIs [6], addition of OFS [8] and addition of CDK4/6 inhibitor abemacilib [9]. These strategies have been shown to reduce the recurrence risk of breast cancer by about 15–35% in different populations. Hence, the significance of detecting CYP2D6 genotype and plasma endoxifen concentrations may not be as crucial as it was before. However, in the population using TAM monotherapy, especially Caucasians or Africans, due to the widespread existence of PM, we assume that endoxifen concentration monitoring is still very important.

The study has some limitations. Firstly, despite a median follow-up duration of nearly 6 years, patients with HR-positive breast cancer remain at risk for late relapse, underscoring the need for extended follow-up periods. And the study was conducted at a single center. Secondly, the PCR method may not be sensitive enough to detect all single nucleotide polymorphisms (SNP). Thirdly, and most importantly, a significant proportion of patients changed their treatment regimen during the follow-up period, which could confound the impact of CYP2D6 genotypes. Finally, concurrent medications were not prospectively collected. Even if we did not find patients taking a strong CYP2D6 inhibitor, the impact might be ignored.

The present study characterized the CYP2D6 genotypes and phenotypes in Chinese patients with breast cancer, and only CYP2D6 IM and NM were identified. All cases had endoxifen concentrations above the threshold of 5.9 ng/ml except for two patients. Endoxifen plasma concentration was higher in patients with CYP2D6 NM compared to IM. No significant differences were observed in adverse reactions between the two groups. These results might have potential implications for further therapeutic options consulting.

## Electronic supplementary material

Below is the link to the electronic supplementary material.

Supplementary Material 1

Supplementary Material 2

## Data Availability

Availability of data and materials Data are available upon reasonable request. Corresponding author: shiyx@sysucc.org.cn.
