# Supplementary material for: CYP2D6 polymorphisms and endoxifen concentration in Chinese patients with breast cancer
Source: BMC Cancer. 2025 Mar 6;25:410. doi: 10.1186/s12885-025-13791-z (PMC11887348; doi:10.1186/s12885-025-13791-z)
Supplement: Supplementary file 1 — Supplementary Material 1 [file 12885_2025_13791_MOESM1_ESM.docx]

1. List of primer sequences and amplified fragments

| SNP | Primers | Amplified fragment size |
| --- | --- | --- |
| rs1065852 | Forward: TCGGTGTGCTGAGAGTGTCCT  Reverse: TGGTTTCACCCACCATCCAT | 355bp |
| rs5030865  rs3892097 | Forward: GGGGAGCATAGGGTTGGAG  Reverse: TGACGTGGATAGGAGGTACAGAGT | 726bp |
| rs16947  rs28371725 | Forward: CGCTTCCAAAAGGCTTTCC  Reverse: GGGGTCCGGCCCTGACACT  Primers for the second round: TGAGGGGAGGCTGGGCAAA | 620bp |
| rs1135840 | Forward: CATGGAGCTCTTCCTCTTCT  Reverse: CAAGGGTAACTGACATCTGC | 360bp |
| *5 | Forward: GTGTCTTTGCTTTCCTGGTG  Reverse: GGTGACTGTTGTGATGGTGC | 506bp |

2. PCR reaction program: 94°C for 5min, (94°C for 30s, 65°C for 30s, 72°C for 30s) for 32cycles, 72°C for 5min, 12°C forever. The amplified products were subjected to agarose gel electrophoresis, and the target fragments were cut out for Sanger sequencing. Sequence analysis was performed by Chromas.

3. CYP2D6 activity Score

| Score | Genotypes | SNP | Activity |
| --- | --- | --- | --- |
| 1 | CYP2D6*1 | - | Normal |
|  | CYP2D6*2 | rs16947(2850C>T) |  |
|  |  | rs1135840(4180G>C) |  |
| 0.5 | CYP2D6*14 | rs5030865(1758G>A) | Reduced |
|  |  | rs16947(2850C>T) |  |
|  |  | rs1135840(4180G>C) |  |
|  | CYP2D6*41 | rs28371725(2988G>A) |  |
|  |  | rs16947(2850C>T) |  |
|  |  | rs1135840(4180G>C) |  |
| 0.25 | CYP2D6*10 | rs1065852(100C>T) |  |
|  |  | rs1135840(4180G>C) |  |
| 0 | CYP2D6*4 | rs3892097(1846G>A) | Lost |
|  |  | rs1065852(100C>T) |  |
|  | CYP2D6*5 | Exon 9 deletion |  |
