# Supplementary material for: CYP2D6 polymorphisms and endoxifen concentration in Chinese patients with breast cancer
Source: BMC Cancer. 2025 Mar 6;25:410. doi: 10.1186/s12885-025-13791-z (PMC11887348; doi:10.1186/s12885-025-13791-z)
Supplement: Supplementary file 2 — Supplementary Material 2 [file 12885_2025_13791_MOESM2_ESM.docx]

Table S1 Adverse events.

| Adverse events | IM (n=46) | NM (n=64) | *p* |
| --- | --- | --- | --- |
|  | N (%) | N (%) |  |
| Liver disfunction events | 14 (30.4) | 22 (34.4) | 0.686 |
| ALT increased | 4 (8.7) | 10 (15.6) | 0.388 |
| AST increased | 5 (10.9) | 14 (21.9) | 0.201 |
| ALP increased | 3 (6.5) | 2 (3.1) | 0.648 |
| GGT increased | 10 (21.7) | 13 (20.3) | >0.999 |
| TBIL increased | 2 (4.3) | 0 (0) | 0.173 |
| Dyslipidemia events | 31 (67.4) | 48 (75) | 0.399 |
| Hypertriglyceridemia | 19 (41.3) | 27 (42.2) | 0.443 |
| Hypercholesteremia | 10 (21.7) | 9 (14.1) | 0.799 |
| HDL increased | 16 (34.8) | 23 (35.9) | >0.999 |
| LDL increased | 19 (41.3) | 20 (31.3) | 0.316 |
| Gynecological events | 16 (34.8) | 23 (35.9) | >0.999 |
| Endometrial thickening | 5 (10.9) | 6 (9.4) | >0.999 |
| Uterine fibroids | 10 (21.7) | 17 (26.6) | >0.999 |
| Ovarian cysts | 5 (10.9) | 6 (9.4) | >0.999 |
| Cervical canal cysts | 6 (13.0) | 10 (15.6) | 0.789 |

Abbreviations: IM, intermediate metabolizer; NM, normal metabolizer; ALT, alanine transferase; AST, aspartate aminotransferase; ALP, alkaline phosphatase; GGT, glutamyl transpeptidase; TBIL, total bilirubin; HDL, high density lipoprotein; LDL, low density lipoprotein.
